# Case of Muscular Atrophy

**Published:** 1871-10

**Authors:** V. Kersey

**Affiliations:** Richmond, Ind.


### SELECTIONS.

### CASE OF MUSCULAR ATROPHY.

By V. KERSEY, M. D., of Richmond, Ind.

Henry C. D., one of the proprietors of the extensive woolen mills recently burnt in this city, was above medium size, of fine form, well developed muscular system, excellent constitution, general good health, and active habits. He was forty years old, a great smoker, and sometimes drank a glass of ale.

In an effort to save some of his property from the fire on the 9th of November, 1870, he and three other men were moving a heavy machine from the lower story of the building. At the door, where they had to ascend three steps, the other men all got in front of the load and he alone behind it, when notice was given that the bell, situated directly above, was about to fall. This led to his lifting up at a great disadvantage, and carrying up the steps a load quite beyond his strength. On getting out of danger, he found himself suffering from acute pain in the right side, and was forced to lie down. After a little rest, however, he recovered so much as to resume work, and continue at it all night. He was very actively employed and very much exposed to inclement weather for several weeks immediately after the fire; during which he had a troublesome cough, a poor appetite, and suffered a constant sensation of intolerable weariness in his right shoulder and arm, and before Christmas he found that the superficial muscles about the shoulder were wasting, and that they were disturbed by irregular twitching, especially when exposed to cold.

The suffering and the cough subsided as the local muscular debility increased; and regarding the latter as a trifling rheumatic affection, he continued his active habits during the early part of winter, using little beyond local applications to the shoulder, measures to promote appetite and digestion, and occasionally the induced electrical current. A little later he was forced to give up active exercise on account of great shortness of breath, which had been growing on him for weeks. From this time he was mostly in the house until his death, which occurred on the 14th June, 1871. I took charge of the case on the 2d of June, twelve days before his death. Except occasional advice from a medical friend, for which he visited a neighboring city several times in the early part of his trouble, he had been under constant homœopathic treatment, and was induced to believe that whenever the doctor got him sufficiently reduced, there would be no trouble or doubt about restoring him to full health and vigor again.

I found him free from pain, with normal sensibility in all parts of his body—able to walk about the room without difficulty, except shortness of breath, which, on a little too much exertion, was very urgent, amounting to almost perfect apnœa.

On careful examination, the lungs, heart, and great vessels were found free from signs of disease. A slight, perhaps simply normal bronchial secretion, gave rise to great annoyance, on account of complete inability to cough, or make the ordinary exertion of clearing the throat. The pulse was about natural in all respects when the patient was quiet, but under exertion it increased in frequency with the approaching apnœa, when the surface became cold, dusky, or motley, and bathed in perspiration. The appetite was generally fair, and the digestion good. The bowels were never moved without artificial means; apparently simply because the diaphragm and abdominal muscles were too much debilitated to afford any aid in defecation. The abdomen was greatly retracted, and the diaphragm highly arched. The function of the kidneys was normal, and the discharge of urine unembarrassed to the last. The sleep was laborious and dreamy, always on the back, with the chin drawn down and the head back, pulling the mouth wide open; and the æration was always worse than during wakeful repose. The mind was generally entirely clear and sound; but sometimes the hallucinations of sleep were held to be real for half an hour or more after waking; though they always ultimately gave way under the better æration of the waking state. The man could not move the right arm except feebly toward the body when the elbow was partially raised. Its muscles and those of the scapula were greatly wasted. The forearm and hand were now fleshy, and on fixing the elbow he could turn them about and use them with some force. The flexors on his limb were less atrophied than the extensors, making it difficult to open the hand, although there was considerable power to grip.

At this stage of the investigation I had the patient sit upright, with the body bare to the seat. From behind the view was remarkable on account of the prominence of the bones on the right side. The muscles on the scapula, both above and below its spine, appeared to be wanting; the shoulder was flattened from atrophy of the deltoid; in a word, all the visible muscles on that side of the trunk were greatly wasted; while on the opposite side the same destructive process was clearly in progress, though not so far advanced. In front the same condition obtained, though not so strongly marked. The left arm seemed to have twice the flesh of the right. Both lower extremities were atrophied to a sensible degree, and here the right side took the lead as above. The muscles concerned in expression, mastication, deglutition, and speech, appeared to be unaffected; their functions were well performed, and no twitching or quivering of their fibres was at any time observed: whereas in parts notably affected with atrophy this was an almost constant symptom, capable, however, of being greatly augmented by exposure of the contiguous surface to a current of cold air. Although the diaphragm and the intercostal and abdominal muscles were less open to observation, the symptoms lead to the conviction that they were among the greatest sufferers from atrophy. The entire inability to cough, sneeze, clear the throat, or promote defecation; the imminent difficulty of respiration; and finally the manner of the death, from simple apnœa, without a struggle, ten minutes after walking from one room to another, seemed to justify this conviction.

Although I had never seen a case of progressive muscular atrophy, I was at once forced to a full recognition of the disease in this man. In the few days he was in my care he was seen by Drs. Walker, Weist, and Clark, who, I believe, fully concurred as to the nature of the disease, though no one of them had seen it before.

I left a small section of muscle from the thick portion of the right pectoralis mayor, with Dr. Weist, requesting a microscopic examination and report. The Doctor kindly furnished the following: "A large number of the muscular fibrillæ—perhaps three-fourths—are entirely normal in appearance save in color, being unusually pale. Transverse striæ very distinct.

"In a portion of the fibrillæ the transverse striæ are very obscure, while in a smaller number they are entirely absent. In the latter the centre of the fibrillæ consists of fine granular matter, evidently exceedingly minute fat globules.

"In a few instances the sarcolemma is collapsed, and almost empty, containing here and there a small oil globule.

"No interstitial deposit of oil globules exists.

"The muscle is evidently undergoing fatty degeneration."

Notwithstanding the apparently hopeless condition of the patient, I advised friction, shampooing, stimulating embrocations, passive exercise of debilitated parts—and, inasmuch as electricity is the only remedy to my knowledge brought forward with a view to the direct arrest of atrophy; and as Faradization had been employed, with another view, in the early weeks of the disease, and without success, I had a wire brought from a Grove Battery of fifty cups, at the telegraph office near by; and after cautious trial, subjected him to the full force of the continuous current. This was kept up from half an hour to an hour, two or three times a day, to the last. His muscular system was sensibly affected by this current, and he had a vague impression that he was benefited by it, and was hopeful of final cure. There was, however, no evidence that it did any good.

With a faint hope of replacing atrophy by nutrition, I prescribed the following pill: R. Sulphate of quinine; lactate of iron; lactate of manganese, of each thirty grains; strychnia crystals; arsenious acid, of each one grain; to be made into mass and divided into forty pills. One to be taken after each meal.

The patient was encouraged to take full rations of good food, including milk, eggs, bread and butter, beefsteak, beef-tea and essence, with tea, coffee, or ale at pleasure.

If there was no mistake in the diagnosis of this case, it presents two considerations worthy of note. The exciting cause of progressive muscular atrophy is generally held to be obscure; in many cases it seems to be wholly unknown. It is sometimes attributed to great muscular exertion; and sometimes to protracted exposure to cold. This man was subjected to the extreme of both these agencies, when, for years, little accustomed to either, and the initial stage of the disease followed rapidly on the exposure.

Again, the disease is generally remarkable for the slowness of its progress—the duration, so far as I know, of recorded fatal cases being a full year at the least, and of others, several years. Here the death occurred seven months and five days after the exposure, and about six months after the probable access of atrophy. Hence this process must have gone forward with unexampled rapidity. But the early fatality was undoubtedly due to the atrophy of a single group of muscles, upon the integrity of which the strictly vital function of respiration depends.—*Proc. Ind. State Med. Soc.*

